# Effect of dislocation slip on in-situ tensile fracture of vanadium alloys after helium/self-ion irradiation

**DOI:** 10.1080/14686996.2026.2627678

**Published:** 2026-02-11

**Authors:** Qianqian Zhang, Shaoning Jiang, Yanfen Li, Shoushuai Zhang, Pengfei Zheng, Jianghai Lin, Guangchun Xiao

**Affiliations:** a School of Mechanical Engineering, Shandong Key Laboratory of CNC Machine Tool Functional Components, Qilu University of Technology (Shandong Academy of Sciences), Jinan, China; b Shandong Institute of Mechanical Design and Research, Jinan, China; c CAS Key Laboratory of Nuclear Materials and Safety Assessment, Institute of Metal Research Chinese Academy of Sciences, Shenyang, China; d Southwestern Institute of Physics, Chengdu, China

**Keywords:** V-4Cr-4Ti, irradiation, in-situ tensile testing, fracture mechanism, dislocation slip

## Abstract

Research on tensile fracture of vanadium alloys after irradiation would help evaluate their mechanical properties and service life in extreme environments of fusion reactors, thereby ensuring the safety and reliability of the materials. This study investigated the effect of dislocation slip on the fracture of irradiated V-4Cr-4Ti through in-situ tensile testing under transmission electron microscopy (TEM). The results showed that He^+^ and V^+^ ions irradiation of V-4Cr-4Ti generated dislocations loops and helium bubbles. During subsequent in-situ tensile deformation, dislocation slip was the primary deformation mode under tensile load, while helium bubbles, due to size constraints, exhibited no significant deformation. During the loading process, multiple slip systems were activated. Based on the Critical resolved shear stress (CRSS) analysis, the slip primarily occurred along the (1 −1 0) plane, while fracture mainly occurred along the (−1 −1 2) plane. The necking phenomenon after fracture was not apparent, indicating that the irradiated sample had a very high yield stress. Additionally, when the sample was close to fracture, dislocations in regions with fewer defects experienced less resistance and thus slid over greater distances.

## IMPACT STATEMENT

This study utilizes in-situ tensile testing under TEM to observe dislocation slip interacts with defects during deformation of irradiated V-4Cr-4Ti, revealing its influence mechanisms on fracture behavior of material.

## GRAPHICAL ABSTRACT

**Figure:**
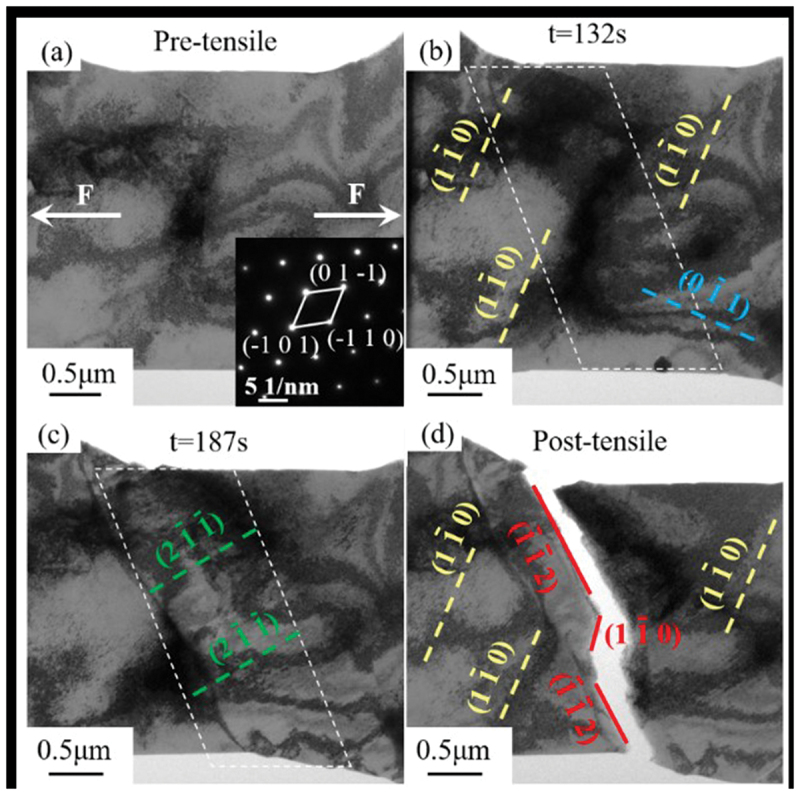

## 1. Introduction

V-4Cr-4Ti, due to its low activation, high-temperature strength, and good compatibility with liquid lithium, was one of the important candidate materials for the blanket structure in fusion reactors. However, vanadium alloys were subjected to high-energy particle irradiation such as neutron and He at high temperature, leading to the formation of a large number of irradiation defects, including point defects, dislocation loops, bubbles and so on [1,2]. Under irradiation, the defects generated inside the material caused lattice distortion and dislocation pinning effects. Although the evolution of these defects did not directly trigger plastic deformation, it significantly increased the ductile-to-brittle transition temperature and reduced ductility, making the material more prone to brittle fracture under thermal stress and leading to the degradation of its mechanical properties [3–6]. Ultimately, this decline diminished the service life of the blankets. Therefore, the deformation and fracture behavior after irradiation were key factors to influence the service condition of vanadium alloys. However, mechanical behavior that was independent of microstructural details did not adequately describe the deformation and fracture behavior. The analysis of dynamic deformation process needed to combine with microstructure evolution, especially for structural materials in blankets, as the irradiation defects might have promoted the formation or movement of dislocation loops.

In recent years, TEM equipped micro-tensile test, i.e. in-situ tensile test made it possible to observe this combinatorial phenomenon [7]. Efforts to understand the slip of dislocation lines had been made, ranging from previous molecular dynamics simulations (MD) [8–11] to current in-situ tensile experiments [12–17]. Previous studies on the tensile fracture mechanisms under non-irradiated conditions had extensively discussed the role of dislocation slip in influencing deformation and fracture behavior. For example, in T92 steel, single-crystal silicon, and nickel-based superalloys, dislocation motion and the activation of slip planes had largely controlled crack initiation and propagation [18–20].

However, when materials are exposed to irradiated environments, tensile fracture mechanisms would undergo significant changes [21–24]. Irradiation produces defects (such as dislocation loops, bubbles, and vacancies) within materials, and the presence of these defects would cause materials to exhibit fracture mechanisms different from those of non-irradiated materials during deformation. For instance, in Fe9Cr1.5W0.4Si F/M steel, the defects such as dislocations introduced by irradiation would impede dislocation movement, leading to the decline of material performance or even failure [25]. Another study indicated that in irradiated austenitic stainless steel, the interaction between dislocations and irradiation-induced defects made crack formation and propagation easier, thereby accelerating fracture behavior [26]. Under fission neutron irradiation conditions, vanadium alloys exhibited significant changes. Fission neutron irradiation not only generated defects within vanadium alloys [4], but also induced irradiation hardening effects, thereby increasing the material’s strength [27]. As the irradiation dose increased, the plasticity of vanadium alloys decreased significantly, ultimately affecting their overall mechanical properties and fracture behavior [28]. Studies also showed that the presence of obstacles under stress affected the slip of dislocation lines and led to both single and multiple cross-slip events [29]. The existence of multiple slip systems complicated the understanding of how dislocation slip influences fracture mechanisms [30,31], and obstacles with larger size made slip more difficult [14]. In areas with fewer defects, slip was easier [15]. Vanadium alloys have a typical Body-Centered Cubic (BCC) structure. Unlike Face-Centered Cubic (FCC) and Hexagonal Close-Packed (HCP) materials, BCC materials have 48 slip systems due to their unique lattice structure [26], resulting in complex and diverse slip mechanisms and slip planes. This makes it challenging to characterize its slip behavior under different conditions. In recent years, studies on BCC metals had provided a basis for understanding complex slip behavior [27–29].

In extreme conditions, such as high temperature, high stress, and high neutron flux, vanadium alloys, as candidate materials for blanket structures, have to maintain excellent mechanical properties while enduring irradiation damage. Some progress has been made in studying the effects of dislocation slip on material properties under irradiation, including pure metals, alloys, single crystals, and polycrystals [29,32,33]. The results found that dislocations were easily influenced by temperature and irradiation defects [34], complicating their motion. Therefore, it was of great importance to further investigate the influence of dislocation slip on the fracture mechanisms in irradiated vanadium alloys. Conducting an in-depth study of the tensile fracture mechanisms of irradiated vanadium alloys was essential for evaluating their mechanical performance and longevity in extreme environments, ultimately ensuring the safety and reliability of materials in nuclear reactors.

In this study, V-4Cr-4Ti alloys were irradiated with a sequence of He ions and V/self-ions at 773 K to simulate actual service conditions, followed by in-situ tensile deformation at room temperature. Macroscopic and microscopic perspectives helped to understand the impact of dislocation slip on fracture mechanisms in V-4Cr-4Ti under stress in near-service environments. This study systematically investigated the dislocation slip behavior of V-4Cr-4Ti after irradiation through in-situ TEM tensile experiments, analyzed the interactions between defects and their effects on crack nucleation. By uncovering the relevant microscopic mechanisms, the research provided important insights for predicting mechanical behavior of V-4Cr-4Ti under high irradiation doses and complex stress conditions in China Fusion Engineering Test Reactor (CFETR) environment.

## 2. Materials and methods

### 2.1. Ion irradiation

The V-4Cr-4Ti alloy used in this study was supplied by the Southwestern Institute of Physics and fabricated via a physical metallurgy process [35]. The preparation began with vanadium-titanium magnetite as the raw material. Ingots were first produced through a vacuum electron beam melting (EBM) process, incorporating a reduction reaction using coke or aluminum as a reductant. The as-cast ingots then underwent multiple subsequent processing steps, including multi-pass vacuum hot die forging followed by five-stage normalization forging. Afterward, the material was vacuum-sealed and subjected to a heat treatment at 1050°C for 30 minutes. This was followed by five passes of hot rolling. It was then cold rolled at room temperature with a reduction of 95%. Finally, a solution treatment was conducted at 1100°C for 1 hour under vacuum atmosphere. The resulting material was V-4Cr-4Ti alloy, which was subsequently cut into samples measuring 5 × 5 × 0.5 mm for further analysis.

For surface preparation, the samples were first mounted using hot-melt adhesive, then sequentially polished with sandpapers of grit sizes 1000, 1500, 2000, 3000, and 5000. Mechanical polishing was subsequently performed using an MP-2A polishing machine with a 0.05 μm SiO_2_ solution as the polishing medium, until the surface was free of visible scratches. Finally, the samples were ultrasonically cleaned in acetone.

**Figure 1.**
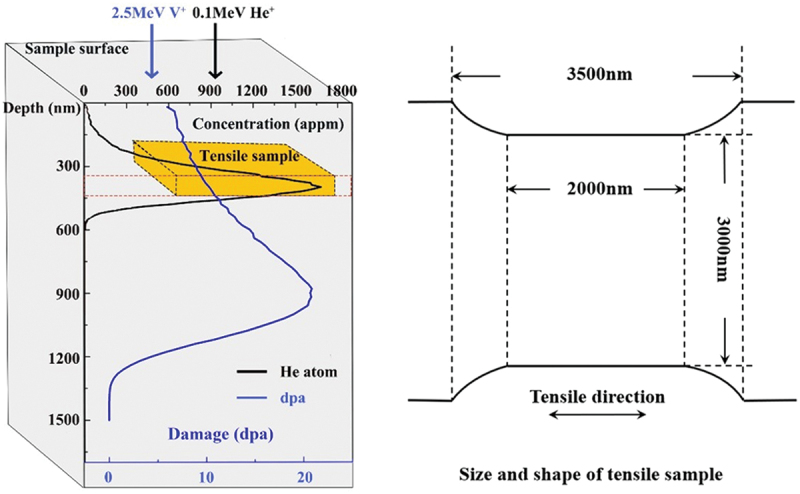
Schematic diagram of the sampling location and dimensions of the tensile samples.

The irradiation condition was decided based on the first-phase plan of CFETR to achieve near-service conditions [36]. He ions were used to simulate the effects of helium in fusion reactors, while self/V (V^+^) ions were used to simulate the defects produced by neutron irradiation, avoiding the additional lattice distortions and chemical effects that other heavy ions might introduce. Sequential He^+^ and V^+^ ion irradiation experiments were conducted on the surface-treated samples using the ion accelerator at Wuhan University. The experiments were carried out at a temperature of 773 K and a vacuum level of 2 × 10^−4^ Pa, with energies of 0.1 and 2.5 MeV, respectively. The fluences of He^+^ and V^+^ were 2.12 × 10^15^ ions/cm^2^ and 1.51 × 10^16^ ions/cm^2^. The irradiation beam flux was 7 × 10^11^ ions/s·cm^2^. Stopping and Range of Ions in Matter (SRIM) software was used to calculate concentration and damage, primarily through two modes: the ‘Full simulation’ mode and the ‘Quick calculation’ mode. The ‘Full simulation’ mode simulates all collision events sequentially, which often yields overestimated damage values. The ‘Quick calculation’ mode handles cascade collisions analytically through fitted approximations and only performs detailed simulations for primary collisions. Therefore,the ‘Full simulation’ and ‘Quick calculation’ modules were used to simulate the ion concentration distributions and damage profiles [37], respectively. The peak concentration of He was 1600 appm, and the total damage was 20 dpa [38]. The damage-depth curve is shown in Figure 1.

### 2.2. In-situ tensile test

After irradiation, the TEM tensile samples were prepared using a focused ion beam (FIB, 30 kV, Thermo Scientific Scios 2) equipped with a Ga ion source and an Omniprobe manipulator. To avoid additional defects affecting the statistical analysis of dislocation rings, a carbon protective layer was first deposited on the surface, and a process with gradually decreasing energy and current was applied. The specific process flow was as follows: 1). Thinning process: The voltage was started at 30 kV and current at 2.5 nA, gradually reduced to 0.23 nA and 80 pA; 2). Polishing process: A voltage of 5 kV and a current of 41 pA were used for polishing; 3). Amorphous layer removal: 2 kV voltage was applied sequentially, using currents of 23 pA and 10 pA for treatment to ensure that the final TEM sample had a clean surface without an amorphous layer [39,40]. To ensure that the stress distribution was as uniform as possible and the deformation behavior was as representative as possible, the sample size was optimized based on the requirements of existing research [41–43], equipment, and the sample’s own characteristics. The final thickness of the tensile sample was 90 nm, located approximately 350 nm from the sample surface, covering the helium peak concentration and the half-peak damage region. The sampling location and dimensions of the tensile samples are illustrated in Figure 1. The choice of half-peak damage region with a damage value of 10 dpa was to avoid the influence of surface effects and self-interstitial effects [44]. The slope of the half-peak region was gentler than that of the peak region, which could maximize the avoidance of damage differences caused by varying depths and reduce the characterization errors of defects resulting from these depth variations.

After the tensile samples were prepared, the morphology was first examined using TEM. The overall morphology was observed using in bright-field (BF) mode. Given the low concentration of helium atoms in the samples, helium bubbles were examined using under-focus and over-focus modes, with focal values of ±1 μm. Selected area electron diffraction (SAED) was used to determine the grain orientation. The g-3 g mode was used for dislocation characterization. In displacement control mode, in-situ quantitative tensile testing was performed in the TEM using a Thermo Fisher (FEI) system. The tensile rate was 10 nm/s. The tensile test process was recorded on video.

## 3. Results

### 3.1. Helium bubbles and dislocation loops in irradiated V-4Cr-4Ti

**Figure 2.**
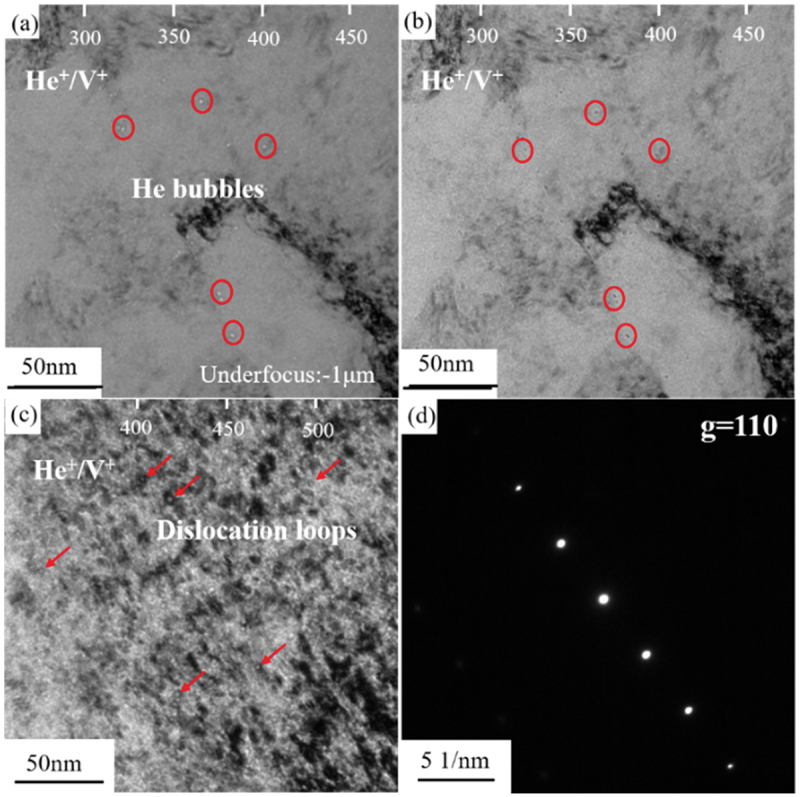
The distribution of helium bubbles and dislocation loops in V-4Cr-4Ti samples after sequential irradiation with He^+^ and V^+^; (a) under-focused image; (b) over-focused image; (c) the microstructure of dislocation loops under g-3 g mode; (d) electron diffraction spots under g-3 g mode.

In V-4Cr-4Ti irradiated with He^+^ and V^+^ ions, He bubbles and dislocation loops were observed, As shown in Figure 2. The bubbles were spherical, with an average size of 4.2 nm and a number density of 3.7 × 10^21^ m^−3^. The dislocation loops had a size of 13.4 nm and a number density of 7.9 × 10^22^ m^−3^. In previous studies conducted by this research group, V-4Cr-4Ti was irradiated with different ions, and Table 1 summarizes the defect information in V-4Cr-4Ti under various irradiation conditions.

**Table 1.** Statistics of bubbles and dislocation loops on V-4Cr-4Ti under different conditions.

Sample	Bubbles		Dislocation loops/dislocation	
	Size (nm)	Number density (m^−3^)	Size (nm)	Number density (m^−3^)
He^+^/V^+^ [45]	4.2	3.7 × 10^21^	13.4	7.9 × 10^22^
V^2+^ [46]	–	–	11	1.63 × 10^23^
He^+^	2.8	5.4 × 10^21^	5.94	5.3 × 10^22^
Unirradiated	–	–	2.64	6.8 × 10^20^

### 3.2. Deformation process and fracture behavior

**Figure 3.**
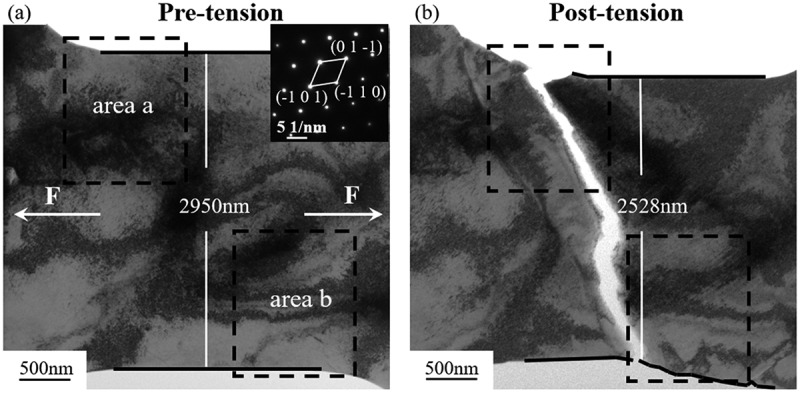
Microstructures of the tensile sample: (a) before stretching; (b) after fracture.

Figure 3(a,b) showed the images before and after in-situ tensile test. Based on SAED analysis before stretching, the loading direction was approximately along [1 0 1], indicated by white arrows in Figure 3(a). Before stretching, the gauge length of the sample was 2950 nm, which decreased to 2528 nm after fracture, a reduction of 14.3%. Misalignment also occurred, as indicated by the black solid line in Figure 3. Fracture mainly occurred along the (−1 −1 2) plane. The fractured sample did not exhibit significant necking or plastic deformation, which was attributed to the irradiated sample having very high yield stress and stable plastic flow [41]. This was similar to the post-fracture behavior observed in austenitic stainless steel and single-crystal nickel materials after irradiation [12,47]. In material tensile experiments, the appearance and propagation of cracks typically affected the final fracture [48].

**Figure 4.**
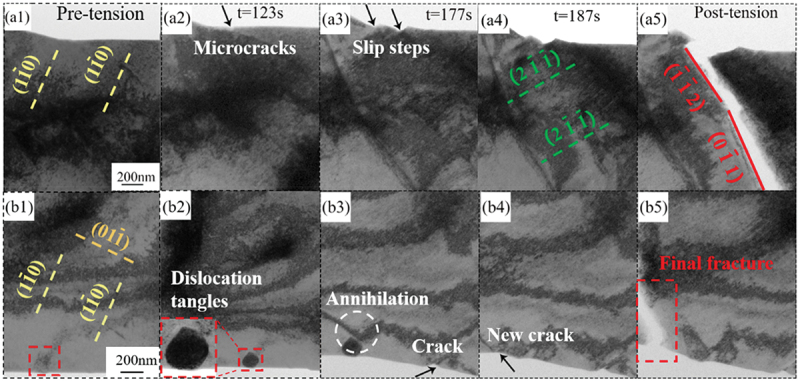
Deformation mechanisms of the tensile samples in regions a and b; (a1) to (a5) area a; (b1) to (b5) area b.

Therefore, the formation and propagation of cracks were analyzed, focusing on two areas, a and b (Figure 3(a)). The tensile deformation mechanisms of the two areas are shown in Figure 4.

Figure 4(a1–a5) and (b1–b5) showed the microstructural evolution and deformation process in area a and b, respectively, from Figure 3. Throughout the tensile process, the movement of dislocations could be clearly observed. Therefore, during the in-situ TEM tensile process, dislocation movement was likely a major factor leading to crackformation [15], with the final fracture occurring at *t* = 188 s. The formation and propagation of cracks were first observed in area a. As shown in Figure 4(a1,a2), during the uniform deformation stage of V-4Cr-4Ti, many dislocations slid along the (1 −1 0) plane. A large number of slipping dislocations accumulated at the edges of the tensile sample, leading to the formation of microcracks, as indicated by black arrowsin Figure 4(a2). These microcracks would become the source of sudden fracture. As the tensile process continued, the cracks further propagated. Before fracture, dislocations escaped from the fracture area, slid along the (2 −1 −1) plane perpendicular to the crack direction, and created slip steps at the edges of the sample, as shown in Figure 4(a3,a4). With continuous loading, the tensile sample ultimately fractured along the (−1 −1 2) and (0 −1 1) slipplanes at 188 s, as shown in Figure 4(a5).

In area b, it was found that the choice of crack propagation appeared to lack a sequence. At the initial stage of loading, dislocation slip occurred along the (1 −1 0) and (0 1 −1) directions, as shown in Figure 4(b1). With the application of tensile loading, a large number of dislocation tangles were observed at the sample edge, and this area was locally magnified, as indicated by the red dashed box in Figure 4(b2). At 177 s of loading, the accumulation of dislocations at the sample edge preferentially led to crack formation. However, the earliest crack did not propagate immediately. In the sample, dislocations gradually evolved into slip bands, generating shear stress in the area where cracks are located, which promoted the slip bands to move towards the dislocation entanglement region, as indicated by the white dashed line in Figure 4(b3). As the tensile load further increased, dislocation entanglement nodes gradually interacted with dislocations in other regions, leading to annihilation to some extent, while stress concentration began to shift to other areas. At a load of 187 s, new cracks reformed at the stress concentration near the edge of the sample, as indicated by the arrows in Figure 4(b4). By 188 s, the earlier cracks had still not propagated, while the newly formed cracks began to expand rapidly under continuous stress, ultimately leading to the overall fracture of the sample. Before fracture, some dislocations slipped in a direction perpendicular to the crack and moved away from the crack region, which was consistent with the observations in area a.

The interaction between dislocations and defects led to the formation of dislocation tangles, increasing the degree of local stress concentration in the material, as shown in Figure 4(b2). As tensile deformation continued, the stress in the material further increased, leading to the formation of slip bands. The movement of these slip bands altered the local stress field [49], which might have resulted in the re-selection of the crack propagation path. As the slip bands continued to move, they partially dispersed the stress at the crack tip, slowing down the propagation of the initially formed crack and promoting the nucleation of new cracks in other regions of high-stress concentration within the material [50]. The further evolution of slip bands led to stress concentration at the forefront of the new crack, while defects such as dislocations and bubbles also accumulated at the crack front, promoting crack propagation. This made crack growth more likely to occur, eventually leading to the macroscopic fracture of the material. It is indicated that the crack propagation path did not always follow the order of crack formation but was more dependent on the nature of the stress field in which the crack was located [30].

Dislocation slip was observed during the stretching process. In the regions of crack propagation, dislocations primarily moved along the (1 −1 0) and (2 −1 −1) planes. By observing the changes during the stretching process, three stages of dislocation slip were identified: the initial stage, the crack propagation stage, and the near-fracture stage.

**Figure 5.**
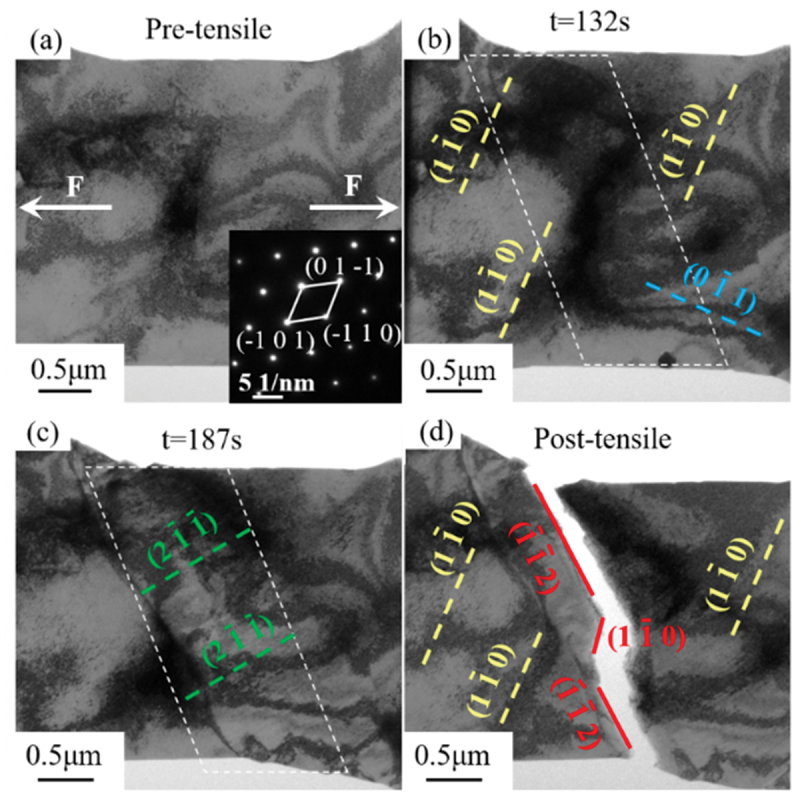
Evolution of dislocations in V-4Cr-4Ti after irradiation during stretching; (a) before stretching began; (b) at 132 s into stretching; (c) at 187 s into stretching; (d) after stretching was complete.

During the initial stage (0–132 s), dislocation slip occurred primarily along the (1 −1 0) plane. Most dislocations in the sample underwent slip, leading to an increase in dislocation density, as shown in Figure 5(a,b). With further loading, cracks first formed at the edges perpendicular to the stretching direction, as shown in Figure 5(b). After the formation of cracks, the slip transitioned into the crack propagation stage (132–187 s). During this phase, in the crack region (marked by the white dashed box), dislocations slipped along a direction perpendicular to the crack, while dislocations in regions farther from the crack nearly ceased to move. This phenomenon continued until the sample was close to fracture. Detailed observations could be seen in Video S1. As the sample approached fracture (187–188 s), dislocations rapidly slipped along the (1 −1 0) plane again, with the process occurring over a short period of approximately 1 s. Additionally, dislocations in regions with fewer defects experienced less resistance and thus slipped over greater distances.

## 4. Discussion

### 4.1. Activation of dislocation slip systems

The in-situ tensile test clearly revealed that dislocation slip was the dominant mechanism for final fracture in the irradiated V-4Cr-4Ti. The experimental results demonstrated that the activation and evolution of dislocation slip systems exhibited a significant stress-dependence and directly influenced crack formation, propagation paths, and the final fracture behavior.

During the initial stage (0–132 s), dislocations primarily underwent relatively short−range slip on the (1 −1 0) planes. Their movement was readily pinned by irradiation-induced defects (e.g. dislocations and bubbles), leading to impeded slip and an increase in dislocation density [51]. As the stress increased, multiple potential slip systems were activated to accommodate plastic deformation and redistribute stress [52–54]. This cooperative action of multiple slip systems promoted an increase in slip band density. However, once the slip band density reached a certain level, the entanglement effects between dislocations (e.g. Figure 4(b2)) significantly intensified, increasing the resistance to dislocation motion and manifesting as material hardening [55]. During the crack propagation stage (132–187 s), particularly in the high-stress concentration region near the crack tip where the stress gradient was steep, dislocations were driven to slide preferentially along specific slip planes (e.g., (2 −1 −1)) from the high-stress zone (crack tip) towards low-stress zones and annihilate. This was a direct manifestation of the strong influence of the stress gradient on dislocation motion direction [42]. In contrast, dislocation activity was less pronounced in regions farther from the crack, where the stress gradient was gentle. Entering the near-fracture stage (187–188 s), plastic deformation gradually transitioned to being dominated by a single slip system (e.g., (1 −1 0)). This likely stemmed from the selective hindering effect of irradiation defects (dislocations and bubbles) on dislocation motion across different slip planes [41], allowing the more adaptable slip system to dominate in the final stage of deformation.

BCC metals were primarily deformed by dislocation slip at room temperature, which was the main mechanism of plastic deformation [56]. The {1 1 0}, {1 1 2}, and {1 2 3} planes were common slip planes in BCC crystals. The activation of dislocation slip systems was closely related to CRSS at the crack tip, which determined the fracture behavior of the material. CRSS was one of the necessary conditions for dislocation slip in materials. When the shear stress decomposed from an externally applied load reached the CRSS on a slip plane in the crystal, dislocation slip began. The magnitude of CRSS in different slip systems determined the deformation mechanism of the material. The CRSS of the slip system could be calculated using the following formula:

(1) $$\tau_{c}=\sigma_{s}\times \mathrm{SF}$$

Where $\tau_{c}$ represented the CRSS magnitude, $\;\sigma_{s}$ represented the yield stress, SF represented the Schmid factor. The condition for the slip system to begin sliding was:

(2) $$\tau \geq \tau_{c}$$

Where, τ represented the shear stress. From Equation (1), it could be seen that the value of CRSS was related to the material’s yield strength and the SF. When τ reached the critical value $\tau_{c}$, the slip systems within the material were activated, leading to plastic deformation of the material.

The SF was calculated using the following equation [46]:

(3)  SF=cosθ×cosψ

**Figure 6.**
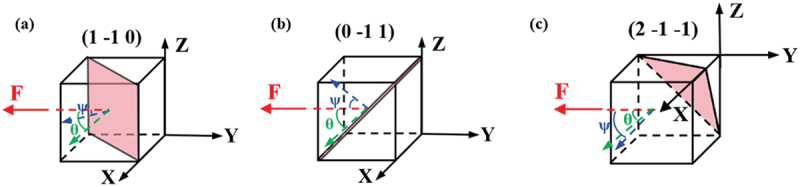
Systematic slip map during the tensile process.

Where, θ was the angle between the loading direction and the slip direction, and ψ was the angle between the loading direction and the normal to the slip plane. The slip systems during the tensile process are illustrated in Figure 6.

During the tensile process, two primary slip systems were mainly observed: (1 −1 0) [1 1 1] and (0 −1 1) [−1 1 1], which were marked with yellow and blue in Figure 5(b), respectively. (2 −1 −1) [1 1 1] was identified as a secondary slip system, marked with green in Figure 5(c). The calculated SF for the slip systems are shown in Table 2 [57].

**Table 2.** Activated slip systems and SF during the in-situ tensile process.

Slip systems	(1 -1 0) [1 1 1]	(0 -1 1) [−1 1 1]	(2 -1 -1) [1 1 1]
**SF**	0.408	0.384	0.236

Then, the value of CRSS could be estimated from Equation (1) through the SF. The smaller the CRSS value, the easier it was for the slip systems to be activated. By calculating the SF values of all slip systems, the SF ratio between the {1 1 2} and {1 1 0} slip systems was obtained, which was approximately 1.15. From Equation (1), it was known that CRSS _{1 1 2}_ = 1.15 × CRSS _{1 1 0}_, meaning that the {1 1 0} < 1 1 1 > slip system was more easily activated than the {1 1 2} < 1 1 1 > slip system. In this study, the {1 2 3} < 1 1 1 > slip system was not observed during tensile testing, primarily because this slip system had a higher CRSS value, making it difficult to activate at room temperature [58,59]. For unirradiated materials, the activation of slip systems was primarily determined by the material’s crystal structure and external stress, with typically low CRSS values [58,60,61]. In irradiated materials, however, irradiation introduced defects such as dislocation loops and bubbles, which impeded dislocation motion by serving as obstacles that pinned dislocations and altered local stress fields. For example, bubbles acted as rigid barriers to dislocation glide, while dislocation loops interacted elastically with moving dislocations [41,62], promoting loop coalescence or dissolution [14,63]. These interactions ultimately affected the distribution of local stress fields within the material, leading to generally higher CRSS values and a more complex trend of variation [64]. For BCC metals, due to hindering effect of defects on dislocation motion, the CRSS of slip systems in irradiated samples was generally higher than that in unirradiated samples. The calculation of *SF* was included in the literature [65], which provided a quantitative value. Compared to unirradiated sample, it was approximately 80% higher. This widespread increase in CRSS not only directly led to a rise in yield stress, but also fundamentally altered the initiation and coordination mechanisms of plastic deformation. In unirradiated materials, dislocation slip was generally easier, and plastic deformation was relatively uniform. Whereas in irradiated samples, the significant pinning effect of defects on dislocations not only delayed the onset of plastic deformation, but also promoted the localization of deformation, thereby causing the material to become prone to brittle fracture at lower overall plastic strains.

### 4.2. Fracture mechanism

**Figure 7.**
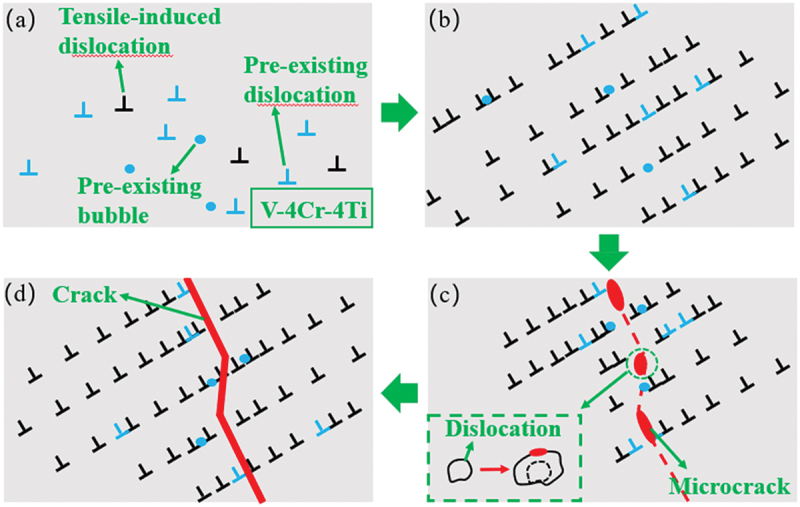
Fracture mechanism diagram induced by dislocation slip.

The evolution of microstructures during in-situ tensile testing showed that irradiation-induced defects impede dislocation slip, leading to dislocation accumulation and local stress concentration, which were the main reasons for the eventual fracture of the material. Figure 7 illustrates the fracture mechanism induced by dislocation slip during the tensile process.

After irradiation, a large number of dislocation loops and a small amount of helium bubbles existed in V-4Cr-4Ti, as shown in Figure 7(a). These irradiation defects, as pre-existing obstacles, had a significant effect on the material’s deformation behavior during the tensile process. During tension, the defects hindered the dislocation slip paths, resulting in a pinning effect. When dislocations encountered these defects during slip, their motion was obstructed, and a large number of dislocations piled up in front of the obstacles (Figure 7(b)). The dislocation pile-up led to a sharp increase in local stress and suppressed plastic deformation through dislocation slip, significantly reducing the material’s ductility and deformability [66].

Notably, the interaction between dislocations and defects described above, along with the resulting degradation of mechanical properties, may be closely related to the microscopic core structure of dislocations themselves. According to the theory of asymmetric core structure, it was known that the asymmetric core structure of dislocations could further modulate the interaction mechanisms between dislocations and irradiation-induced defects, thereby altering the distribution and evolution of local stress fields and ultimately influencing the nucleation and propagation paths of cracks [67,68]. In this study, the observed cracks primarily propagated along (−1 −1 2) plane. This phenomenon likely originated from anisotropy of such slip resistance and the specific interaction patterns between dislocations and defects such as bubbles and dislocation loops.

As tensile stress was continuously applied, dislocations were repeatedly obstructed and moved by obstacles, causing the thickness of the dislocation region to gradually decrease. The local stress field was further enhanced, eventually exceeding the material’s local bearing limit, which became the nucleation point for cracks, triggering the formation of microcracks, as shown in Figure 7(c). The formation mechanism of microcracks mainly involved the activation of multiple slip systems, the motion and accumulation of dislocations, and non-uniform strain at grain boundaries (GBs) [69]. In V-4Cr-4Ti, the interaction between dislocation pile-up and irradiation defects was the primary driving force for microcrack nucleation. When the local stress field in the dislocation pile-up region interacted with irradiation defects such as bubbles, the crystal structure around the defects was further weakened, becoming a preferred path for crack propagation. At the same time, GBs, as defect accumulation centers, also played a key role in crack nucleation and propagation [70]. Due to the accumulation of irradiation defects and local stress concentration in the GB region, microcracks were more likely to initiate near the GBs and extend into the grain interior.

Once microcracks were formed, their tips rapidly propagated due to high-stress concentration. The stress field created by dislocation pile-up, combined with the local stress field around the defects, caused the high-stress region to exceed the material’s fracture strength. Cracks typically appeared at the junctions of dislocation pile-up and defects and expanded outward. When cracks expanded and connected multiple microcracks, they gradually developed into macroscopic through cracks. The crack propagation path was significantly influenced by the distribution of GBs and defects, ultimately leading to the overall failure of the material (Figure 7(d)). Under the irradiation conditions of this experiment, the fracture phenomenon in V-4Cr-4Ti occurred near the peak concentration of He, which corresponded to the damage half-peak region. This phenomenon was primarily attributed to the aggregation of high concentrations of helium atoms in this region, leading to the formation of numerous nanoscale bubbles. These bubbles caused local microstructural embrittlement, thereby significantly reducing the fracture toughness of materials. In addition, it is worth noting that helium-vacancy (He-V) clusters, which were invisible under TEM, also played a critical role in fracture behavior [41,71,72]. These clusters acted as effective microcrack initiation sites within crystal structure, not only promoting the early nucleation and growth of bubbles but also intensifying stress concentration effects, thereby further accelerating crack propagation and the premature failure of materials. Previous studies indicated that the accumulation of dislocation density in polycrystalline materials could also induce the final fracture of the material [73]. Due to the TEM samples are thin, the constraint effect by adjacent GBs is relatively weak. During deformation, thin samples may exhibit a phenomenon of tensile axis (bending). While the bulk samples may be affected greatly by size effects and GBs constraints during deformation [74,75]. Bulk samples, due to their larger volume, have more complex internal GBs constraints, and their deformation mechanisms may also be more diversified. More importantly, regardless of the sample thickness, GBs, as potential weak points or stress concentration areas, and their properties, distribution, and interactions would influence the final fracture locations of the material [76]. The multiple analytical techniques would be used in future to gain a more comprehensive understanding of the deformation differences between thin and bulk samples. The study revealed the interaction mechanisms between partial dislocations and irradiation-induced defects, established the connection between microscopic mechanical properties and macroscopic mechanical properties, and laid a key foundation for accurately predicting and optimizing the service performance of vanadium alloys in extreme environments of fusion reactors.

## 5. Conclusion

After undergoing irradiation experiments under conditions close to service, the V-4Cr-4Ti alloy produced a large number of dislocation loops and a small amount of helium bubbles. In-situ TEM tensile tests were conducted to observe the dislocation slip and fracture behavior of the irradiated samples. The main findings were:

1. The fracture of V-4Cr-4Ti after irradiation was caused by dislocation slip, primarily occurring along (−1 −1 2) plane. The obstruction and pinning effects between irradiation defects and dislocations played a decisive role in the fracture behavior of the material.
2. Under tensile loading, multiple slip systems were activated, including (1 −1 0) [1 1 1]、(0 −1 1) [−1 1 1] and (2 −1 −1) [1 1 1]. The complex changes in the local stress field during the tensile process were the main reason for this behavior.
3. The {1 1 0} < 1 1 1 > slip system was believed to initiate slip first due to its lower CRSS value. This was consistent with the observed slip behavior during in-situ TEM tensile tests.

## Supplementary Material

Supplemental Material

Supplemental Material

## Data Availability

The data that has been used is confidential.

## Statement of novelty

This study utilizes in-situ tensile testing under TEM to observe dislocation slip interacts with defects during deformation of irradiated V-4Cr-4Ti, revealing its influence mechanisms on fracture behavior of material.
