# Supplementary figures and images for: Effect of dislocation slip on in-situ tensile fracture of vanadium alloys after helium/self-ion irradiation
Source: Sci Technol Adv Mater. 2026 Feb 11;27(1):2627678. doi: 10.1080/14686996.2026.2627678 (PMC12943815; doi:10.1080/14686996.2026.2627678)

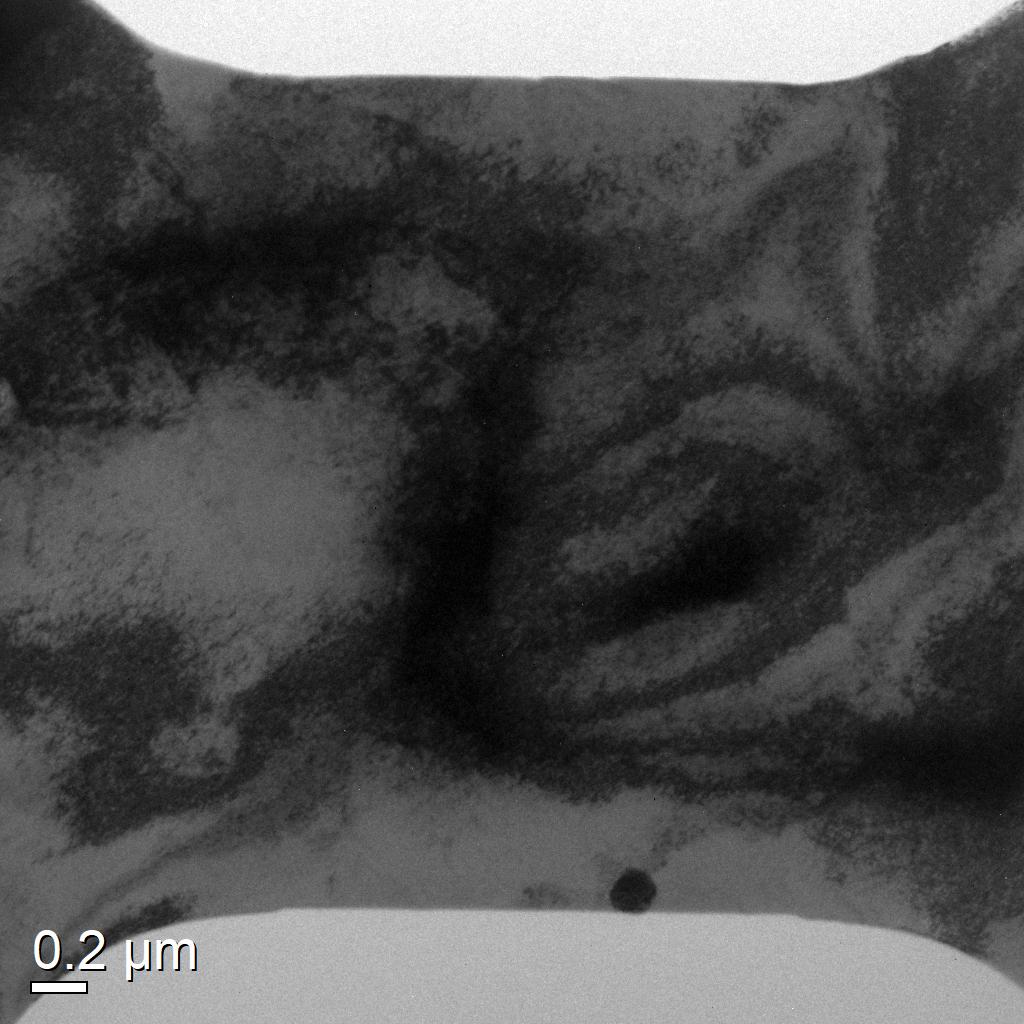

Supplement: Supplemental Material [file TSTA_A_2627678_SM7280.jpg]
